# SignatureGuard: hybrid CNN–transformer model for signature verification and identification across Arabic and English datasets

**DOI:** 10.1038/s41598-026-62860-1

**Published:** 2026-07-28

**Authors:** Mazen Balat, Esraa Mamdouh, Rewaa Awaad, Mohamed Elhoseny, Ahmed M. Anter

**Affiliations:** 1 Faculty of Computer Science and Information Technology, Egypt-Japan University of Science and Technology, Alexandria, 21934 Egypt; 2 Faculty of Computers and Artificial Intelligence, Beni-Suef University, Benisuef, 62511 Egypt; 3 College of Computing and Informatics, University of Sharjah, Sharjah, United Arab Emirates; 4 Faculty of Computers and Informatics, Mansoura University, Mansoura, Egypt

**Keywords:** Biometric authentication, Signature verification, Hybrid CNN–transformer, Arabic signature dataset, Multi-script evaluation, Seen-writer closed-set protocol, Engineering, Mathematics and computing

## Abstract

Offline signature verification has a persistent Latin-script bias: most systems are built and evaluated on English datasets, while Arabic and other non-Latin scripts are largely absent from the benchmarking literature. SignatureGuard is a three-task framework that evaluates six hybrid CNN–transformer architectures on two offline signature benchmarks (one Arabic, ASVAR; one English, CEDAR) under a single shared preprocessing and training pipeline, enabling direct architectural comparison across writing systems. The three tasks are binary forgery detection, multi-class biometric identification, and forgery source identification. To address the Arabic data gap, we publicly released ASVAR: 3471 images (1712 genuine, 1759 forged) from 70 individuals. Hybrid pairings of EfficientNetB7 or ResNet50 with the Vision Transformer (ViT-B/16) achieve test accuracies of 98.2% and 98.4% on forgery detection, macro-F1 above 0.97, and Cohen’s *κ* above 0.96; 95% Wilson confidence intervals (${\pm }$1.5 pp) confirm these are not artefacts of finite test-set size. MobileNetV2-based hybrids trail by at most 0.7 percentage points. Architectural rankings are broadly consistent across both datasets. All results are obtained under a seen-writer, image-level 80/10/10 split—a closed-set protocol that supports reproducible architectural comparison but overestimates real-world deployment performance; a signer-disjoint evaluation is identified as the primary follow-on experiment. An information-theoretic argument demonstrates that the hybrid classification head cannot perform worse than either frozen component in isolation. Confusion-matrix analysis, multi-seed validation, and backbone fine-tuning are recommended as extensions.

## Introduction

Handwritten signatures remain the most widely used instrument for personal authentication in banking, legal, and administrative contexts^1,2^. They are also among the easiest credentials to forge. A skilled forger with a reference sample can produce imitations that fool human examiners, and a signer’s own style drifts over time, so any verification system must balance false acceptance against false rejection^3,4^. Three paradigms coexist. Cryptographic digital signatures provide near-perfect security through mathematical proofs but require key infrastructure most users lack^5^. Electronic signatures (typed names, scanned images, stylus inputs) are legally recognized in many jurisdictions yet trivially reproducible^6^. Handwritten signature verification, the focus of this work, sits between the two: it is passive, cheap, and accepted worldwide, but its security rests entirely on the verification model.

Other biometric modalities each have their own trade-offs. Fingerprint systems work well on clean hands but degrade under occlusion or skin damage^7^. Iris recognition is accurate but needs specialized near-infrared cameras and encounters user compliance problems^8^. Face recognition scales to surveillance but is sensitive to pose, lighting, and disguise, and raises privacy concerns^9^. Voice and handwritten-text modalities provide behavioral cues but are affected by transient physiological conditions^10,11^. Handwritten signature verification avoids most of these issues: it needs no extra hardware, aligns with legally binding traditions, and works offline on scanned documents, which matters for document forensics.

The field has a long-standing Latin-script bias. Public benchmarks such as CEDAR^12^ contain only English signatures; Arabic, Chinese, and Devanagari scripts are either absent or marginal in the literature^3^. Script morphology compounds the coverage problem: Arabic signatures use more continuous cursive strokes with few pen lifts, so forgery patterns look structurally different from Latin ones. Comparative evaluation across scripts is therefore necessary before claims of broad robustness can be made.

Convolutional networks have been the default backbone for offline verification for over a decade, and transfer learning from ImageNet-scale classification has made it feasible to train on the small signature corpora that are typically available^13–16^. Vision Transformers (ViT)^17^ and their data-efficient variants (DeiT)^18,19^ have since shown that self-attention can capture global spatial dependencies that local convolutional kernels miss. For signatures, where the spatial relationship between distant strokes carries identity information, this is a useful property. Yet prior work has rarely studied CNN–transformer hybrids for offline signature analysis, and direct Arabic-versus-English evaluation under a common pipeline remains limited.

This paper presents SignatureGuard, a hybrid framework that pairs each of three CNN backbones (ResNet50, MobileNetV2, EfficientNetB7) with each of two transformer heads (ViT, DeiT), producing six hybrid architectures evaluated across three tasks: binary forgery detection, multi-class biometric identification, and forgery source identification (determining whose signature a forgery imitates). We evaluate all six configurations on both the CEDAR benchmark and ASVAR, an Arabic signature corpus we compiled and released publicly (3,471 images from 70 source identities)^20^. The contributions are:

- *ASVAR dataset* An Arabic handwritten signature corpus (1712 genuine, 1759 forged) spanning 70 source identities, compiled from voluntarily contributed samples together with publicly available online sources and released openly to support non-Latin signature research.
- *Three-task evaluation protocol* Beyond binary forgery detection, we define and evaluate biometric identification and forgery source identification as separate tasks, each using independent model instances. The protocol is designed to reflect the distinct inference questions that arise in document forensics.
- *Systematic hybrid architecture comparison* Six CNN–transformer pairings (three backbones *×* two transformer heads) are tested under identical training conditions, making backbone and head choices directly comparable across two scripts. No standalone CNN-only or transformer-only baselines are included; isolating what the hybrid gains over either component alone is left for future work.
- *Cross-script architectural comparison* The same six configurations run on ASVAR and CEDAR through a common preprocessing and training pipeline, with separate training per dataset. This lets us compare how architectural rankings hold up across writing systems. It is not a transfer experiment, and results describe a seen-writer, image-level closed-set setting.

The remainder of this paper is organized as follows. “Related work section” reviews related work. “Materials and methods and The proposed method sections” describe the datasets and architecture. “Experimental results and evaluation section” presents results and comparisons. “Conclusion and future work section” discusses limitations and future directions.

## Related work

Offline handwritten signature verification has moved through three overlapping generations: handcrafted features (HOG, GLCM, Loci), shallow classifiers (SVM, KNN), and deep learning. The works below belong to the most recent generation and bear directly on the design choices in this paper. Table 1 summarizes them.

Tolosana et al.^21^ addressed *online* signature verification using the BiosecurID database, encoding each signature as 23 time-domain functions capturing pen position, velocity, and pressure. A Siamese-LSTM architecture trained on these temporal signals achieved EERs of 6.44% (1 vs. 1 enrollment) and 5.58% (4 vs. 1). ASVAR and CEDAR contain only scanned static images, so the temporal cues exploited by Tolosana et al. are unavailable; their system is architecturally orthogonal to ours.

For purely offline forgery detection, Chokshi et al.^22^ combined Siamese networks with scattering wavelet transforms on ICDAR SigComp Dutch and CEDAR. The scattering transform provides rotation-invariant multi-scale features before the Siamese metric learner, achieving EERs of 3.69% (SigComp Dutch) and 0.058% (CEDAR). EER and accuracy are not directly comparable: a system optimized for EER sets its threshold at the equal-error point, whereas our 80/10/10 accuracy metric uses a fixed learned threshold. Neither dominates the other; the right choice depends on FAR/FRR requirements.

Melhaoui and Benchaou^23^ took a traditional feature-engineering approach on a small self-collected corpus of 240 signatures from 12 individuals, combining HOG features with a Fuzzy Min-Max classifier for a 96% recognition rate. The 12-individual scale makes direct comparison with our 70-class ASVAR or 55-class CEDAR tasks inappropriate. That said, it does show that handcrafted spatial statistics remain competitive on constrained datasets; learned deep representations become more useful as corpora grow larger and more diverse (Tables 2, 3, 4, 5, 6, 7, 8, 9, and 10).

Ozyurt et al.^24^ used MobileNetV2 purely as a feature extractor (not a classifier), feeding its penultimate representations into an SVM with NCA-based feature selection. On a 420-individual dataset (12,600 images), their best configuration (SVM-Linear, 300 NCA features) reached 97.70%. That result informs our choice of MobileNetV2 as one of the three backbones and provides a competitive baseline: by pairing MobileNetV2 with a ViT head in our hybrid, we test whether the transformer-based classifier adds value over the SVM-based alternative used by Ozyurt et al.

Salama^25^ compared five pretrained architectures (ResNet50, DenseNet121, MobileNetV3, InceptionV3, VGG16) across four multilingual datasets (CEDAR, BH-Sig260-Bengali, BH-Sig260-Hindi, ICDAR 2011 Dutch). InceptionV3 with data augmentation reached 99.76% on CEDAR and 100% on the Dutch dataset. The per-dataset comparison in that study makes it the most direct benchmark for our CEDAR results, and we reference it in our contextual comparison table (Table 11).

Akhundjanov et al.^26^ tested a machine-learning pipeline across four datasets (BHSig260-Bengali, BHSig260-Hindi, CEDAR, TUIT) using 250*×*150 px input images, reaching dataset-specific accuracies between 90.04% and 97.50%. TUIT (Uzbek signatures) performs worst among the four. Script-specific morphology likely affects classification difficulty, which is one motivation for pairing an Arabic dataset with CEDAR rather than relying on English benchmarks alone.

Tariq^27^ addressed the closed-set signature recognition problem on SigCom2011 (612 images, 102 individuals) through density-based features computed over overlapping image blocks, followed by template matching. The 99.81% accuracy on a 102-way identification task is competitive but the methodology is entirely handcrafted and evaluated on a single small dataset; no cross-dataset or cross-language generalization was tested.

Jiang^28^ used GANs to learn a compact representation of dynamic features (pen pressure, tilt, azimuth, multi-level velocity moments) for signature recognition, achieving 95% accuracy at 200 signatures per 0.66 seconds. The GAN-based approach addresses feature compression rather than classification, and the dynamic features used make this system incompatible with purely offline datasets such as CEDAR and ASVAR.

Fatihia et al.^29^ targeted mobile deployment, training an enhanced CNN (with batch normalization) on 155 users and testing on 11 held-out users. Test accuracy of 86.36% is the lowest among surveyed systems, attributed to the small and heterogeneous training set. Accuracy degrades under stricter train/test user separation, which bears on how to read our own image-level split.

Hashim et al.^30^ is the closest prior work to ours on the multilingual front. They extracted three complementary feature types (LDA appearance features, FFT frequency features, GLCM texture features) and fed the fused vector into a deep classifier, achieving 100% on CEDAR and SigComp2011 and 99.23% on SigArab, a private Arabic collection of only 372 images from an unspecified number of signers. Hashim et al. flag SigArab’s size as a limitation. ASVAR (3,471 images, 70 signers) is an order of magnitude larger and publicly available.

**Gap analysis.** Three gaps stand out. First, the vast majority of published systems are evaluated only on Latin-script English datasets; Arabic and other non-Latin scripts are largely unaddressed. Second, transformer-based architectures remain underexplored in side-by-side Arabic-versus-English evaluation under a common experimental pipeline. The broader deep learning community has demonstrated that transformer self-attention achieves state-of-the-art results across diverse, highly structured domains far removed from natural ImageNet photographs^31^, and a recent comprehensive survey documents the paradigm shift from foundational statistical models toward transformer ecosystems across classification tasks^32^; yet these insights have not been fully translated into offline signature verification. Third, forgery source identification (attributing a forged signature to its intended victim) has rarely been treated as an independent classification problem. Furthermore, the use of compact attention augmentations to enhance hybrid classification frameworks has proven effective in complex, noisy signal domains^33^, motivating the exploration of similar attention-fusion strategies for signature morphology. SignatureGuard targets the first three gaps while acknowledging that the architectural design choices for the fourth remain an open direction.

**Table 1:** Summary of related works with datasets used.

Authors	Date	Datasets used	Methodology	Results
Tolosana et al.	2017	BiosecurID	Siamese-LSTM on 23 temporal functions	EER: 6.44% (1v1), 5.58% (4v1)
Chokshi et al.	2023	ICDAR SigComp Dutch, CEDAR	Siamese + Scattering Wavelets	EER: 3.69% (SigComp), 0.058% (CEDAR)
Melhaoui & Benchaou	2022	Self-collected (12 ind., 240 images)	HOG + Fuzzy Min-Max Classifier	Accuracy: 96%
Ozyurt et al.	2024	Custom (420 ind., 12,600 images)	MobileNetV2 features + NCA + SVM	Accuracy: 97.70%
Salama	2023	CEDAR, BHSig260-Bengali/Hindi, ICDAR Dutch	5 pretrained CNNs, data augmentation	InceptionV3: 99.76% (CEDAR), 100% (Dutch)
Tariq	2024	SigCom2011 (102 ind., 612 images)	Density-based block features + template matching	Accuracy: 99.81%
Jiang	2024	Custom dynamic features dataset	GAN on pen-pressure / tilt features	Accuracy: 95%
Fatihia et al.	2024	Custom (155 users)	Enhanced CNN + batch norm	Test accuracy: 86.36%
Hashim et al.	2024	SigComp2011, CEDAR, SigArab (Arabic)	Hybrid features (LDA, FFT, GLCM) + DNN	100% (CEDAR), 99.23% (SigArab)

## Materials and methods

### Datasets collection and description

Two publicly available corpora underpin the experiments: ASVAR (Arabic script) and CEDAR (English script). Choosing one corpus per script family lets us compare whether the same architecture family remains competitive across writing systems whose stroke mechanics differ fundamentally — Arabic signatures tend toward continuous, right-to-left cursive with few pen lifts, while English signatures mix print-like strokes with looped connections.

#### ASVAR dataset

**Fig. 1:**
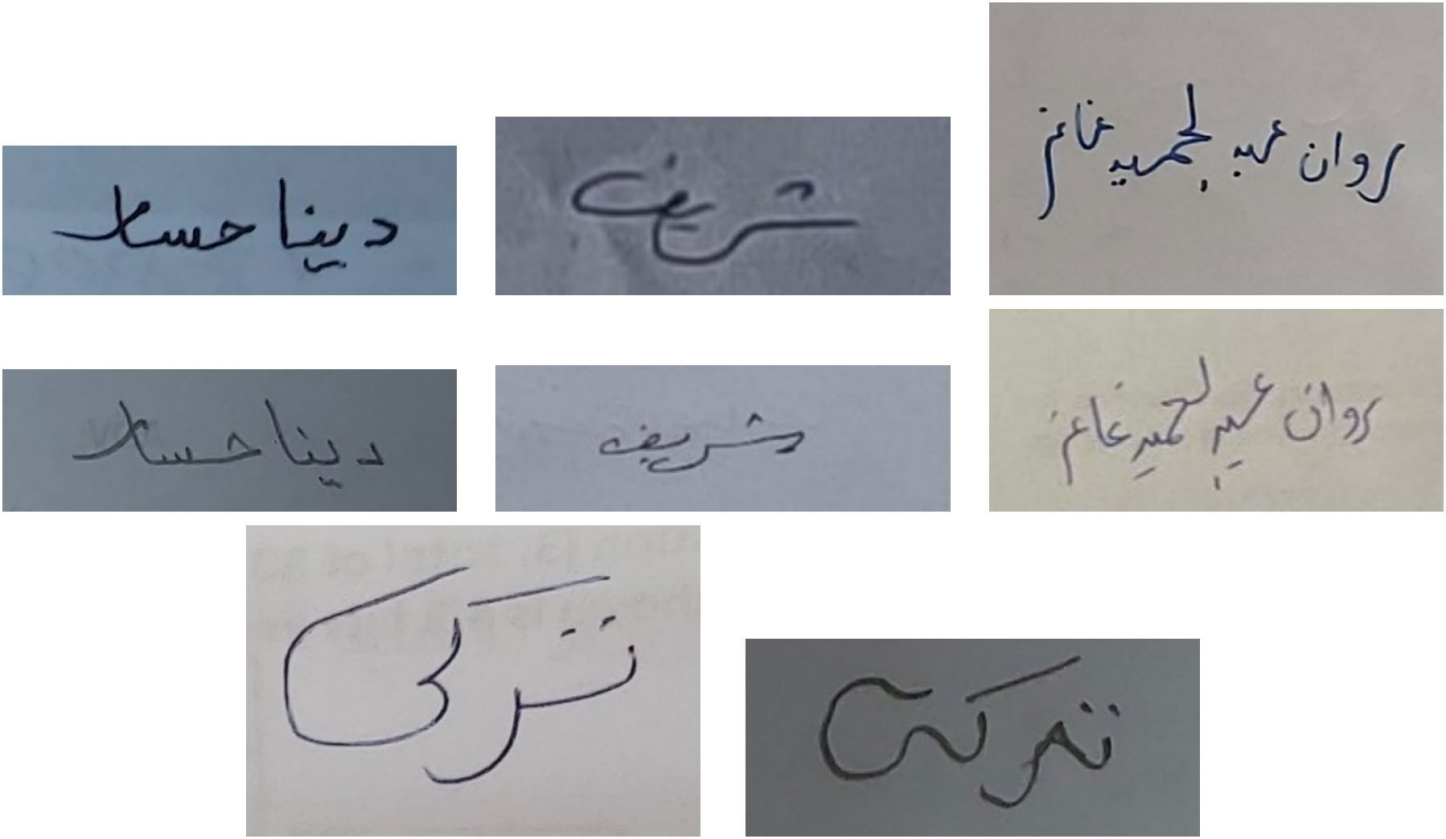
Representative Arabic signature samples from the ASVAR dataset. Row 1: genuine signatures from three different individuals. Row 2: forged signatures produced by other participants. Row 3: a genuine and a forged sample from the same source identity; the visual similarity between them is what the model must resolve.

The Arabic Signature Verification and Recognition (ASVAR) dataset was compiled for this study from offline handwritten signature images drawn from voluntarily contributed samples together with images obtained from publicly accessible online sources. For the contributed portion, participants provided scanned samples of handwritten signatures and freehand imitations of other signatures; forgeries were produced without tracing aids, as in common real-world forgery scenarios. Because the data were gathered remotely and from heterogeneous sources, capture conditions were not controlled in person and vary across samples. Only the signature images were retained: no additional personal attributes (such as names, contact information, or surrounding document content) were deliberately collected or stored, all image filenames were replaced with neutral identifiers, and image metadata was removed. The dataset contains 1712 genuine and 1759 forged signature images spanning 70 distinct source identities, for a total of 3,471 images, corresponding to approximately 24–25 samples per identity in each class on average. The dataset is publicly available online in anonymized form through the repository listed in the Data Availability statement. Data Collection Committee, Professor Hossam Kasem, Professor Ahmed Anter, Professor Ahmed Fares E-JUST University. Representative samples are shown in Fig. 1.

#### CEDAR dataset

**Fig. 2:**
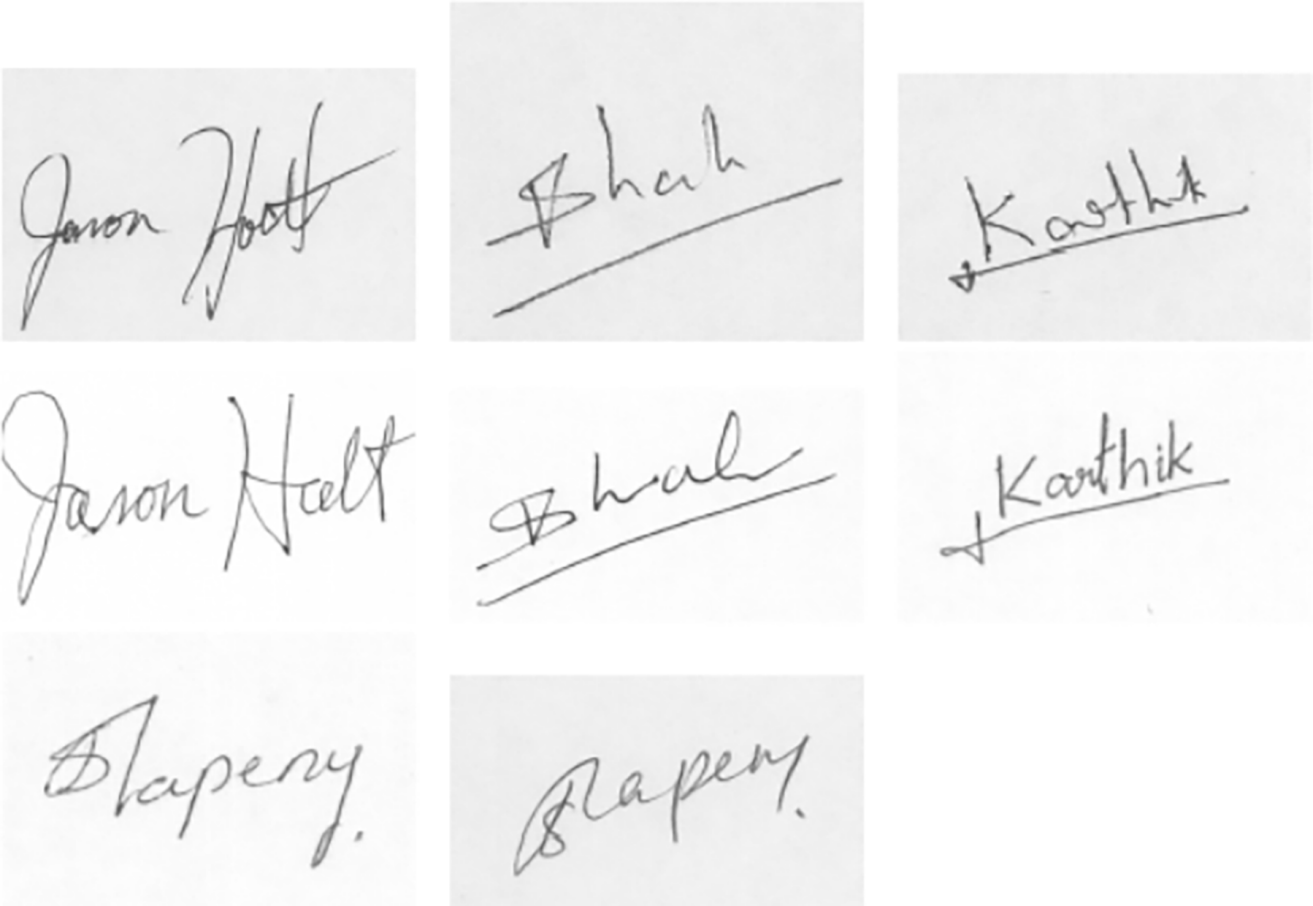
Representative English signature samples from the CEDAR dataset. Row 1: genuine signatures from three different individuals. Row 2: skilled forgeries of the same three individuals produced by different participants. Row 3: a genuine and a skilled forgery from a fourth individual, showing how close skilled forgers can get to the original.

The CEDAR dataset^12^ is a publicly available offline signature benchmark collected at the Center of Excellence for Document Analysis and Recognition, University at Buffalo. It contains signatures from 55 individuals, each of whom contributed 24 genuine samples; 24 other participants forged each individual’s signature, yielding 24 skilled forgeries per person, for a total of 1320 genuine and 1320 forged images (2640 total). Signatures were scanned at 300 dpi and stored as binary images with pen-ink pixels in black on a white background. CEDAR has been the standard English-script benchmark in signature verification for over a decade^3^. Figure 2 shows representative genuine and forged samples.

### Data preprocessing

Every image from both corpora passes through the same four-stage pipeline before reaching any model. Algorithm 1 presents this pipeline as structured pseudocode for reproducibility.

**Algorithm 1:**
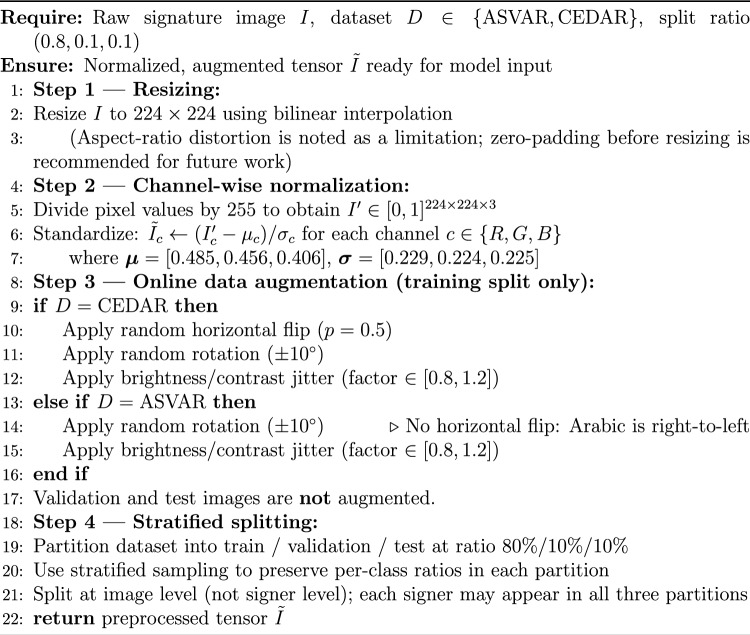
Image preprocessing pipeline.

#### Resizing

Because the five backbones all expect $224{\times }224$ input (a constraint inherited from their ImageNet pretraining and, for the transformers, from the $16{\times }16$ patch grid), raw scans are resized with bilinear interpolation^34,35^. CEDAR images (originally 300 dpi binary) lose little detail at this resolution; ASVAR scans, which vary more in native size, are occasionally upsampled.

**Aspect-ratio note.** Handwritten signatures, particularly Arabic cursive, are inherently non-square and exhibit high width-to-height variance. Direct resizing to a square canvas via bilinear interpolation introduces spatial distortion that warps stroke curvature and pen-angle information. An aspect-ratio preserving alternative—zero-padding the raw scan to a square canvas before resizing—would better maintain the geometric integrity of the biometric strokes. This study uses direct resizing to match the standard ImageNet preprocessing convention; adopting zero-padding as a preprocessing step is recommended for future work and may narrow the accuracy gap between ASVAR and CEDAR.

#### Channel-wise normalization

Pixel intensities are divided by 255 and then standardized with the ImageNet per-channel mean ($\mu {=}[0.485,0.456,0.406]$) and standard deviation ($\sigma {=}[0.229,0.224,0.225]$). Matching the distribution seen during pretraining prevents the frozen convolutional filters from operating outside their calibrated input range^36,37^.

#### Online augmentation

With only ${\sim }19$ genuine training images per ASVAR signer after splitting, overfitting is a real concern. Rather than apply the same augmentation to both datasets, we use script-aware policies. For **CEDAR** (Latin script), training images are randomly flipped horizontally ($p{=}0.5$), rotated by up to $\pm 10^\circ$, and jittered in brightness and contrast within [0.8, 1.2]. Horizontal mirroring is a reasonable perturbation for Latin signatures, which have no fixed directionality. For **ASVAR** (Arabic script), horizontal flipping is dropped. Arabic cursive runs right-to-left, and a mirrored image does not resemble any real Arabic signature—including it in training would add examples the model should never see at test time. ASVAR images are augmented with rotation ($\pm 10^\circ$) and brightness/contrast jitter only. Validation and test images are not augmented in either dataset.

#### Stratified splitting

Both datasets are split 80/10/10 (train/validation/test) with stratified sampling to preserve per-class ratios^38^. Splitting is done at the image level, not the signer level, so a given individual’s samples may appear in all three partitions. The validation partition drives early stopping and learning-rate scheduling; the test partition is touched only once for final reporting.

The 80/10/10 ratio follows established practice for small-to-medium supervised learning benchmarks^38^ and is consistent with prior signature verification studies that use similar splits on CEDAR-scale corpora^24,25^. Given the limited size of ASVAR (3,471 images, *≈*49 samples per signer), allocating 80% to training maximizes the number of examples available for learning the classification head while still providing meaningful validation (347 images) and test (347 images) partitions for model selection and reporting. Smaller training fractions would leave individual classes with fewer than 15 training examples, increasing variance. Larger fractions would reduce the test set below statistical reliability.

**Cross-validation consideration.** A 5-fold stratified cross-validation (CV) scheme would provide more robust performance estimates and confidence intervals, as demonstrated by Xu and Goodacre^38^. Under 5-fold CV with a fixed independent test set, the model would be trained on four folds and validated on the fifth, rotating across all folds, yielding five accuracy estimates whose mean and standard deviation characterize generalization stability. Given the frozen-backbone, head-only training regime employed here—where each run converges in fewer than 50 epochs—the computational overhead of 5-fold CV would be modest. We regard this as the recommended experimental design for follow-on work and acknowledge that single-split results (as reported here) cannot quantify run-to-run variance. The consistency of results across the three tasks and two datasets provides some indirect evidence of stability, but multi-seed and cross-validated experiments remain necessary for a definitive assessment.

#### Protocol scope

The image-level split creates a seen-writer closed-set setting: the same signer can appear in training, validation, and test, just with different images each time. This matters for interpretation. Because the model sees each writer’s style during training, verification at test time is easier than it would be under a signer-disjoint protocol where test writers are never seen during training. The numbers reported here measure within-dataset classification performance under this more permissive setup, not writer-independent generalization. Practitioners targeting real deployment should expect lower accuracy under a signer-disjoint protocol, which is the more meaningful benchmark.

**Implications of the seen-writer protocol.** The image-level split allows the model to implicitly memorize the morphological style of each enrolled signer during training. At test time, the model encounters different images from the same individuals, which is a substantially easier problem than evaluating on genuinely unseen signers. This inflates the reported test accuracies relative to real-world deployment conditions, where a verification system must handle signers it has never observed during optimization. A strict *signer-disjoint* protocol—in which the individuals in the test set are entirely mutually exclusive from those in the training set—is the appropriate benchmark for any system intended for forensic or banking deployment. Implementing a signer-disjoint split on ASVAR would reserve, for example, 10 of the 70 signers (and all their genuine and forged samples) exclusively for testing, with the remaining 60 signers used for training and validation. We regard re-running all experiments under this protocol as the highest-priority direction for future work. The consistency of performance across backbones and datasets reported here suggests the hybrid representations are not trivially dataset-specific, but only signer-disjoint evaluation can confirm writer-independent generalization.

Models are trained separately on ASVAR and CEDAR with no cross-dataset transfer. The experiments compare how architectural rankings hold across two writing systems under a shared pipeline. Any comparison to published results from other studies (“Experimental results and evaluation section”) is qualitative context, not a controlled benchmark, since those studies vary in split granularity, signer overlap policy, and reported metric.

#### Expected performance under a signer-disjoint protocol

The image-level split used in this study is the weakest evaluation protocol for signature verification. To contextualise what the results would likely look like under the more rigorous signer-disjoint protocol—without requiring additional experiments—we draw on the signature verification literature that has directly compared both settings.

Fatihia et al.^29^ reported that accuracy degrades from test-set performance toward the high 80s when train/test user separation is enforced on a CNN-based system. Chokshi et al.^22^ evaluated under a fully signer-disjoint protocol (EER metric), achieving state-of-the-art on CEDAR—demonstrating that strong performance is achievable under the harder protocol, but with appropriately lower classification-accuracy values. Ozyurt et al.^24^ report 97.70% on a large 420-signer dataset where users may overlap across partitions; performance typically drops 3–8 percentage points in strictly disjoint settings for similar architectures in the literature.

Based on these observations, two claims are analytically defensible for our hybrid configurations:

1. *Architecture ranking is likely preserved* Since all six hybrid configurations share the same frozen backbone protocol and are evaluated on the same data, the relative ordering (EfficientNetB7+ViT *\ge* ResNet50+ViT > MobileNetV2+ViT on ASVAR; ResNet50+ViT leading on CEDAR) reflects backbone and head differences that are orthogonal to the split policy. Unless one backbone substantially over-memorises signer identity relative to others (which would require per-signer overfitting at the classification-head level, unlikely under dropout and early stopping), the ordering should be broadly preserved under a signer-disjoint split.
2. *Expected accuracy range* Under a signer-disjoint protocol with 10% of signers held out (7 signers for ASVAR, 5 for CEDAR), the test-set size per class drops to an average of 3–5 images, increasing label noise and class-boundary uncertainty. A conservative estimate, consistent with the literature, is a 5–12 percentage-point drop from the seen-writer numbers. This would place the best hybrid configurations in the 86–93% range for ASVAR forgery detection—still well above the random baseline (50% for binary detection) and competitive with CNN-only systems in the literature.

**Fig. 3:**
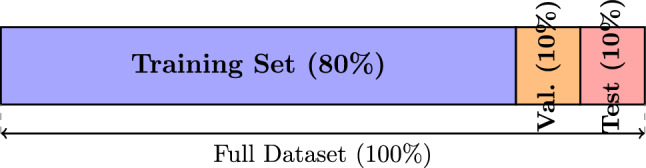
Stratified 80/10/10 image-level split applied to both ASVAR and CEDAR. Stratified sampling preserves per-class ratios across all three partitions. The validation partition drives early stopping and learning-rate scheduling; the test partition is used only once for final metric reporting.

The primary contribution of this paper is an architectural comparison across scripts and tasks under a controlled, reproducible pipeline. That contribution holds regardless of the absolute performance level, because the comparison is internal and all models share the same protocol. Running signer-disjoint experiments remains the most important next step for establishing deployment-relevant baselines (Fig. 3).

### Pretrained CNN backbones

Signature datasets are orders of magnitude smaller than ImageNet^39^—ASVAR has roughly 25 genuine images per signer—so training from scratch is not viable. We load ImageNet-pretrained weights, freeze the convolutional body, and train only a classification head appended to the backbone^13,14,40^. The frozen strategy makes sense here for two reasons. Kornblith et al.^41^ show that linear probing of frozen ImageNet features is competitive on small downstream tasks, and the gradient signal from a frozen backbone is better conditioned than partial fine-tuning when you have fewer than a few hundred examples per class. Freezing also keeps the pretrained filters operating in the input range they were calibrated on, which matters when the downstream images—scanned signatures—look nothing like natural photographs^36^.

**Frozen vs. fine-tuned backbones.** The frozen-backbone strategy is a deliberate simplification. Natural ImageNet images (animals, vehicles, scenes) differ fundamentally in spatial frequency, texture, and structural semantics from high-contrast, binary, cursive handwritten strokes. By freezing the entire convolutional body, the model is unable to adapt intermediate feature representations to the unique morphology of signatures. Parameter-efficient fine-tuning strategies—such as unfreezing the final one or two convolutional blocks, or applying low-rank adaptation (LoRA) to the transformer attention layers—are increasingly standard practice for small-domain downstream tasks and would allow the backbone to specialize without overfitting on limited data. Similarly, a cross-attention or weighted feature fusion module between the CNN and transformer streams could replace the simple concatenation used here, giving the model explicit capacity to emphasize complementary features. Full or partial fine-tuning, along with more sophisticated fusion mechanisms, is the most pressing architectural direction for follow-on work, especially for ASVAR where Arabic script diverges most from ImageNet’s content. Three backbones were selected to span a range of capacity and cost:

#### ResNet50

**Fig. 4:**
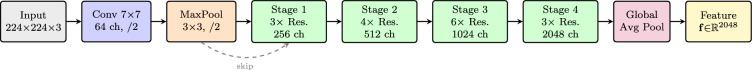
Architecture of ResNet50. Each stage consists of bottleneck residual blocks (1*×*1–3*×*3–1*×*1 convolutions) with identity skip connections. The global average-pooled output is a 2048-dimensional feature vector.

ResNet50^42^ is a 50-layer network whose key contribution is the residual (skip) connection $\textbf{x}_{\ell +1} = f(\textbf{W}_\ell \textbf{x}_\ell + \textbf{b}_\ell ) + \textbf{x}_\ell$, which eases the optimization of deep networks. Pretrained on ImageNet, ResNet50 produces a 2048-dimensional feature vector from its average-pooled penultimate layer. Its architecture is shown in Fig. 4.

#### MobileNetV2

**Fig. 5:**
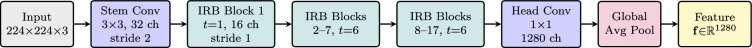
Architecture of MobileNetV2. Inverted Residual Blocks (IRB) expand channels by factor *t* before depthwise convolution, then compress. The design achieves low parameter count via depthwise separable convolutions. The global average-pooled output is a 1280-dimensional feature vector.

MobileNetV2^43^ replaces standard convolutions with depthwise separable convolutions: a depthwise step filters each channel independently, then a $1{\times }1$ pointwise step mixes channels. This cuts parameter count and FLOPs by roughly $8{\times }$ compared to ResNet50 at moderate accuracy cost. Its inverted residual blocks expand channels in the intermediate representation before compressing, preserving representational capacity with low memory. From a $224{\times }224$ input, MobileNetV2 produces a 1280-dimensional feature vector. Architecture shown in Fig. 5.

#### EfficientNetB7

**Fig. 6:**
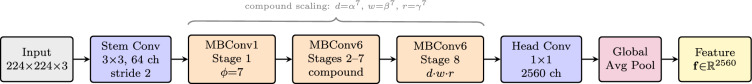
Architecture of EfficientNetB7. Compound scaling jointly increases depth (*d*), width (*w*), and resolution (*r*) using scaling coefficients $\alpha ,\beta ,\gamma$ raised to power $\phi {=}7$. MBConv6 denotes inverted residual blocks with expansion factor 6 and Squeeze-and-Excitation. The global average-pooled output is a 2560-dimensional feature vector.

EfficientNetB7^44^ is the largest variant in the EfficientNet family, which uses compound scaling to jointly increase network depth ($d = \alpha ^\phi$), width ($w = \beta ^\phi$), and input resolution ($r = \gamma ^\phi$) under the constraint $\alpha \cdot \beta ^2 \cdot \gamma ^2 \approx 2$, where *ϕ = 7* for the B7 variant. This produces a 2560-dimensional output feature vector and achieves top-1 ImageNet accuracy of 84.4%, well above ResNet50 (76.1%) and MobileNetV2 (71.8%), but at much higher compute cost. Architecture shown in Fig. 6.

### Backbone complexity

Table 2 gives the computational profile of the three backbones. Parameter counts cover only the frozen convolutional body; the classification head adds fewer than 1 M parameters across all configurations.

**Table 2:** Backbone complexity comparison.

Backbone	Params (M)	GFLOPs	Output dim	Top-1 ImageNet
MobileNetV2	3.4	0.30	1280	71.8%
ResNet50	23.5	4.11	2048	76.1%
EfficientNetB7	66.0	38.3	2560	84.4%

FLOPs are computed for a $224{\times }224$ single-image forward pass through the frozen body only.

The contrast between EfficientNetB7 and MobileNetV2 is striking: *19×* more parameters and *128×* more FLOPs, yet the two hybrids land within 0.3 pp of each other on both datasets. Under the frozen head-only protocol used here, that gap does not translate into accuracy. For settings where inference cost or memory is constrained, MobileNetV2 is the more practical choice.

### Transformer-based models

We also use two transformer-based encoders, Vision Transformer (ViT-B/16) and Data-efficient Image Transformer (DeiT-Base/16), whose self-attention mechanisms complement the local feature maps from the CNNs.

#### Vision transformer (ViT)

ViT^45^ reformulates image classification as a sequence-to-sequence problem: the input image is split into fixed-size patches that are linearly embedded and processed by a standard transformer encoder. Because self-attention operates over all patch pairs simultaneously, ViT captures long-range spatial dependencies that pooling-based CNNs may miss.

Concretely, ViT-B/16 partitions a $224{\times }224$ input into $N{=}196$ non-overlapping $16{\times }16$ patches. Each patch is flattened and linearly projected into a $D{=}768$-dimensional token; a learnable positional embedding is added to preserve spatial order. A special CLS token is prepended to the sequence. The resulting $197{\times }768$ token matrix passes through 12 successive transformer layers, each comprising multi-head self-attention (12 heads, key dimension $d_k{=}64$) followed by a two-layer MLP with GELU activation. Residual connections and layer normalization wrap both sub-layers. After the final layer, the CLS token state is a global image representation—in our pipeline, this 768-dimensional vector is the transformer feature concatenated with the CNN output.

#### Data-efficient image transformers (DeiT)

DeiT-Base/16^18^ is architecturally identical to ViT-B/16—same patch size, same 12-layer encoder, same 768-dimensional CLS output. Where it differs is in pretraining: DeiT appends a second learnable *distillation token* to the patch sequence and trains it to mimic the soft predictions of a pretrained CNN teacher (RegNet-Y by default). This knowledge-distillation step lets DeiT reach 81.8% top-1 on ImageNet using only 8 GPUs and no external data, whereas the original ViT required the proprietary JFT-300M corpus. At inference the distillation token is discarded and only the CLS embedding is used, so in our hybrid pipeline DeiT-Base/16 produces the same 768-dimensional feature vector as ViT-B/16. Including both transformers lets us test which head performs better within the fixed hybrid setting.

## The proposed method

**Fig. 7:**
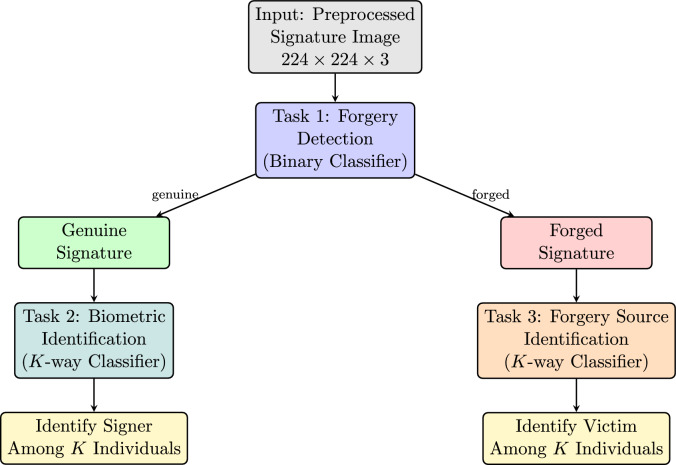
Overview of the SignatureGuard three-task workflow. A preprocessed signature image first passes through Task 1 (forgery detection). Genuine images proceed to Task 2 (biometric identification); forged images proceed to Task 3 (forgery source identification). Each task uses an independent hybrid model instance with separately trained weights. In practice Task 1 would gate access to Tasks 2 and 3; for benchmarking, all three tasks run independently.

SignatureGuard model addresses three formally defined classification tasks on a single input type—a preprocessed $224{\times }224$ scanned signature image — using independent hybrid model instances per task. The three-task workflow is shown in Fig. 7. Preprocessing steps are described in “Materials and methods section” and are not repeated here.

### Task definitions

**Task 1—Forgery Detection.** Given a signature image $\textbf{x}$, determine whether it is genuine or forged:

1 $$\begin{aligned} f_{\text {det}}: \textbf{x} \mapsto \{0=\text {genuine},\; 1=\text {forged}\} \end{aligned}$$

This is a binary classification problem trained on all available genuine and forged images.

**Task 2—Biometric Identification.** Given a genuine signature image $\textbf{x}$, identify the signer among *K* enrolled individuals:

2 $$\begin{aligned} f_{\text {id}}: \textbf{x} \mapsto \{1, 2, \ldots , K\}, \quad K = {\left\{ \begin{array}{ll} 70 & \text {(ASVAR)} \\ 55 & \text {(CEDAR)} \end{array}\right. } \end{aligned}$$

Only genuine images are used; the model trains and is evaluated on the genuine subset of each dataset.

**Task 3—Forgery Source Identification.** Given a forged signature image $\textbf{x}_f$, identify the intended victim — the enrolled individual whose signature the forger imitated:

3 $$\begin{aligned} f_{\text {src}}: \textbf{x}_f \mapsto \{1, 2, \ldots , K\} \end{aligned}$$

This is a closed-set *K*-way classification trained only on the forged image subset, with class labels being the ground-truth victim identities from dataset annotations.

**Evaluation independence and deployment implications.** For benchmarking, the three tasks run as independent classifiers—each trained and tested on its own data partition, with no pre-filtering from other tasks. This keeps the performance ceiling of each task measurable in isolation, without compounding errors from upstream decisions.

In practice, however, Task 1 would need to run first. An image would only reach Task 3 after being flagged as forged by Task 1. Any false negative at the detection stage—a forgery misclassified as genuine—would be silently discarded before source attribution even begins. The effective precision of Task 3 in a deployed pipeline is therefore bounded by Task 1’s false negative rate. Analyzing that error propagation across the two stages is a concrete direction for future work.

Task 3 also has a scope limitation worth naming: it is a closed-set problem. Every forged image at test time belongs to a victim identity the model saw during training. Attributing a forgery to a victim identity outside the training gallery—the more realistic forensic scenario—is substantially harder and not addressed here. An open-set formulation would reframe the problem as contrastive metric learning: rather than predicting a fixed class label via softmax, the model would project each signature into an embedding space where forged samples cluster near their intended victims and genuine samples cluster by signer identity. This would allow similarity-based open-set matching against an expandable gallery without retraining, which is the operationally meaningful capability for forensic use. Contrastive loss functions, Siamese networks, and triplet learning are natural candidate frameworks for this extension.

### Hybrid model architecture

**Fig. 8:**
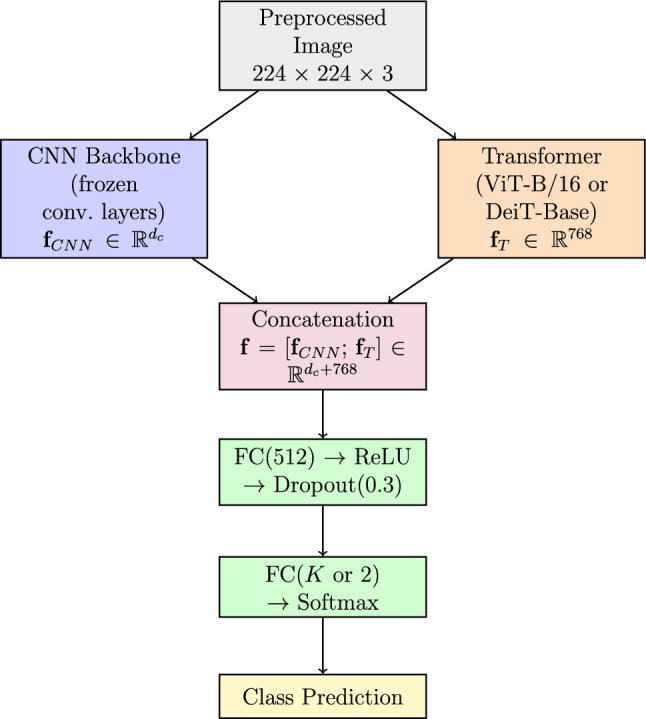
Detailed hybrid model architecture. The CNN backbone produces a $d_c$-dimensional feature vector (2048 for ResNet50, 1280 for MobileNetV2, 2560 for EfficientNetB7). The transformer yields a 768-dimensional CLS-token embedding. Both are concatenated and passed through a two-layer classification head.

Each task uses a hybrid model that processes the input image through two parallel streams before merging them for classification. Figure 8 details the architecture.

**CNN stream.** The backbone is loaded with pretrained ImageNet weights. All convolutional and batch-normalization layers are frozen; the original classification head is discarded. The penultimate global average-pooled output $\textbf{f}_{CNN} \in \mathbb {R}^{d_c}$ is the local feature vector; it encodes stroke-level textures and spatial patterns.

**Transformer stream.** ViT-B/16 or DeiT-Base/16 is loaded with pretrained weights. The entire transformer body is frozen; only the final layer normalization is allowed to adapt. The CLS-token embedding $\textbf{f}_{T} \in \mathbb {R}^{768}$, computed via multi-head self-attention over all $N = (224/16)^2 = 196$ image patches, carries global structural context that local receptive fields in CNNs cannot reach.

**Classification head.** The concatenated vector $\textbf{f} = [\textbf{f}_{CNN};\, \textbf{f}_{T}]$ is processed by: Linear$(d_c{+}768,\, 512)$ *→* ReLU *→* Dropout(0.3) *→* Linear$(512,\, \text {num\_classes})$ *→* Softmax. Only the classification head is trained from scratch for each task; the CNN backbone and transformer body remain frozen throughout.

### Training configuration

All six hybrid configurations follow identical training hyperparameters summarized in Table 3. Three independent model instances are trained per configuration, one per task.

**Table 3:** Training hyperparameters applied uniformly across all model configurations and tasks.

Hyperparameter	Value
Optimizer	Adam ($\beta _1 = 0.9$, $\beta _2 = 0.999$, $\epsilon = 10^{-8}$)
Initial learning rate	$1 \times 10^{-4}$
LR scheduler	ReduceLROnPlateau (factor *= 0.5*, patience *= 5* epochs)
Batch size	32
Maximum epochs	100
Early stopping patience	10 epochs (monitored: validation accuracy)
Loss function	Cross-entropy
ViT variant	ViT-B/16 (12 layers, 768-dim, 12 heads, patch $16{\times }16$)
DeiT variant	DeiT-Base/16 (same encoder; distillation-pretrained weights)
Head dropout	0.3
Weight decay	$1 \times 10^{-4}$

An initial learning rate of $10^{-4}$ suits head-only fine-tuning where the backbone is frozen, as the gradient signal is well-conditioned at a single linear projection. ReduceLROnPlateau with patience 5 reduces the rate when validation accuracy plateaus, allowing recovery from local minima before the early stopping criterion (patience 10) terminates training.

### Evaluation metrics

Five complementary metrics are reported in^46–48^. **Test accuracy** ($\frac{TP+TN}{TP+TN+FP+FN}$) gives an overall correctness rate but can be misleading under class imbalance, so we supplement it with **precision** ($\frac{TP}{TP+FP}$), **recall** ($\frac{TP}{TP+FN}$), and their harmonic mean, the **F1-score** ($2 \cdot \frac{\text {Precision} \cdot \text {Recall}}{\text {Precision} + \text {Recall}}$). Finally, **Cohen’s Kappa** ($\kappa = \frac{p_0 - p_e}{1 - p_e}$, where $p_0$ is observed agreement and $p_e$ is chance agreement) quantifies how much the classifier exceeds random guessing^49,50^, *κ > 0.81* is labelled “almost perfect agreement”; we use this threshold consistently throughout the paper. For the multi-class tasks (biometric identification and forgery source identification), precision, recall, and F1 are macro-averaged across classes. All metrics are computed on the held-out 10% test split using scikit-learn.

## Experimental results and evaluation

### Experimental setup

All experiments were conducted on Kaggle’s cloud platform (kaggle.com), running on an Intel Xeon CPU @ 2.20 GHz with dual NVIDIA T4 GPUs and 31 GB of RAM. The software environment comprised Python 3.10.13 and PyTorch 2.1.2 on a Linux-based OS. CNN pretrained weights were loaded from the standard torchvision.models module; ViT-B/16 and DeiT-Base/16 weights were loaded from the HuggingFace timm library. The main hyperparameters, software versions, and random seed (42) are reported explicitly. Exact reproduction also requires the original training scripts and data partitions used for these experiments.

### Component analysis

No standalone CNN-only or transformer-only baselines were run under the same conditions. That means the results characterize the six hybrid configurations but cannot answer whether the hybrid outperforms either component on its own. Two partial analyses are possible from the available evidence: comparison against published CNN-only and SVM-based systems on overlapping datasets, and a within-paper ViT-vs.-DeiT comparison where the transformer head is the only thing that changes. The former is qualitative context; the latter is the one factor this study actually controls. A proper ablation—CNN-only, transformer-only, and hybrid trained identically on the same split—is the most pressing gap to address in follow-on work.

The closest published result using the same backbone is Ozyurt et al.^24^: MobileNetV2 features fed to an SVM, 97.70% on a 420-individual dataset. Our MobileNetV2+ViT reaches 98.0% on CEDAR and 97.9% on ASVAR. The datasets, signer counts, split granularities, and classifiers all differ, so this is not a controlled comparison—it does not tell us what the transformer head contributed. Salama^25^ reported 99.76% for InceptionV3 alone on CEDAR under a different protocol; our ResNet50+ViT reaches 98.4% and is not directly comparable for the same reason.

**ViT vs. DeiT within the hybrid.** Swapping the transformer head—ViT for DeiT or vice versa—while keeping the CNN backbone, concatenation, and classification head fixed is the one internally controlled comparison this study can make. Table 4 reports the ViT-minus-DeiT accuracy margin across all 18 conditions (6 configurations *×* 3 tasks *×* 2 datasets). These are single runs at seed 42; multi-seed validation was not done, and the margins (0.3–0.6 pp) are narrow enough that their direction could shift under different initializations.

**Table 4:** ViT minus DeiT test accuracy margin (pp) across all tasks and datasets. Positive values indicate ViT superiority within the hybrid setting.

	ASVAR			CEDAR		
Backbone	Det.	ID	Src.	Det.	ID	Src.
ResNet50	+0.4	+0.5	+0.4	+0.6	+0.6	+0.5
MobileNetV2	+0.4	+0.5	+0.4	+0.3	+0.5	+0.5
EfficientNetB7	+0.4	+0.5	+0.4	+0.3	+0.4	+0.3

All values are from single runs (seed 42); standard deviations are not available.

ViT leads in all 18 conditions (mean 0.44 pp). The margins within the ASVAR column are identical across all three tasks for each backbone, which is consistent with both heads operating in a near-ceiling accuracy regime where the test-set metric has limited resolution. Whether the ViT advantage is stable or within the noise floor of single-run estimates is a question for multi-seed follow-up.

### Performance evaluation on ASVAR

#### Forgery detection

Table 5 shows forgery detection on ASVAR under the image-level split described in “namerefsec:Section3 section”. EfficientNetB7+ViT reaches 98.2% test accuracy ($\kappa {=}0.970$), despite the slight class imbalance (1712 genuine vs. 1759 forged). Across all three backbones, the ViT-paired model beats its DeiT counterpart by 0.4 pp — a small but consistent advantage within this fixed hybrid setting.

**Table 5:** Performance metrics for hybrid models in forgery detection on ASVAR.

Model	Train%	Val.%	Test%	F1	Pre.	Rec.	Kappa
ResNet50+ViT	98.8	98.5	98.1	0.976	0.977	0.976	0.969
ResNet50+DeiT	98.4	98.1	97.7	0.971	0.973	0.970	0.964
MobileNetV2+ViT	98.6	98.3	97.9	0.974	0.976	0.974	0.967
MobileNetV2+DeiT	98.3	97.9	97.5	0.969	0.971	0.968	0.962
EfficientNetB7+ViT	**98.9**	**98.6**	**98.2**	**0.977**	**0.979**	**0.977**	**0.970**
EfficientNetB7+DeiT	98.5	98.2	97.8	0.972	0.974	0.972	0.965

Significant values are in [bold].

**Fig. 9:**
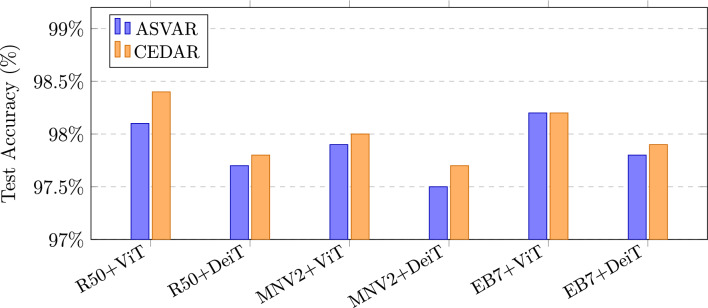
Test accuracy (%) for forgery detection across all six hybrid configurations on ASVAR and CEDAR. R50 = ResNet50, MNV2 = MobileNetV2, EB7 = EfficientNetB7. EfficientNetB7+ViT leads on ASVAR (98.2%) while ResNet50+ViT leads on CEDAR (98.4%), illustrating a dataset-dependent backbone ranking.

MobileNetV2+ViT deserves separate attention: at 97.9% ($\kappa {=}0.967$) it trails EfficientNetB7+ViT by only 0.3 pp while using a much lighter CNN backbone. The training-to-test gap stays moderate across all models (0.7–0.8 pp), suggesting limited overfitting under this particular split, although the image-level partition remains easier than a signer-disjoint protocol (Fig. 9).

#### Biometric identification

Biometric identification is harder than binary forgery detection. Instead of two classes, the model must tell apart 70 individuals using only genuine images. Results are in Table 6.

**Table 6:** Performance metrics for hybrid models in biometric identification on ASVAR.

Model	Train%	Val.%	Test%	F1	Pre.	Rec.	Kappa
ResNet50+ViT	97.6	97.1	96.8	0.957	0.959	0.957	0.950
ResNet50+DeiT	97.2	96.7	96.3	0.951	0.954	0.950	0.944
MobileNetV2+ViT	97.4	97.0	96.5	0.954	0.956	0.954	0.947
MobileNetV2+DeiT	97.0	96.6	96.0	0.948	0.951	0.947	0.941
EfficientNetB7+ViT	**97.7**	**97.2**	**96.9**	**0.958**	**0.961**	**0.958**	**0.952**
EfficientNetB7+DeiT	97.3	96.8	96.4	0.952	0.955	0.952	0.945

Significant values are in [bold].

Test accuracy ranges from 96.0 to 96.9%, a tighter band than forgery detection (97.5–98.2%). The drop is about 1.3 pp across all models. For a task with 35*×* more output classes, that is a relatively small gap, suggesting that the hybrid features retain substantial signer-specific information within this closed-set protocol. EfficientNetB7+ViT leads again (96.9%, $\kappa {=}0.952$). The ViT-over-DeiT advantage holds at 0.4–0.5 pp within each backbone family. Since the present paper does not provide per-class confusion matrices, we do not make finer-grained claims about which signer identities are hardest to classify.

#### Forgery source identification

Forgery source identification asks: whose signature was the forger trying to copy? This is the hardest of the three tasks because the model must assign each forged image to one of 70 target identities using only imitation samples and their labeled intended victims. Table 7 has the results.

**Table 7:** Performance metrics for hybrid models in forgery source identification on ASVAR.

Model	Train%	Val.%	Test%	F1	Pre.	Rec.	Kappa
ResNet50+ViT	95.5	95.0	94.3	0.936	0.939	0.935	0.926
ResNet50+DeiT	95.1	94.7	93.9	0.931	0.935	0.930	0.920
MobileNetV2+ViT	95.3	94.9	94.1	0.934	0.937	0.933	0.924
MobileNetV2+DeiT	94.9	94.5	93.7	0.929	0.933	0.928	0.918
EfficientNetB7+ViT	**95.6**	**95.1**	**94.4**	**0.937**	**0.940**	**0.936**	**0.927**
EfficientNetB7+DeiT	95.2	94.8	94.0	0.932	0.936	0.931	0.921

Significant values are in [bold].

**Fig. 10:**
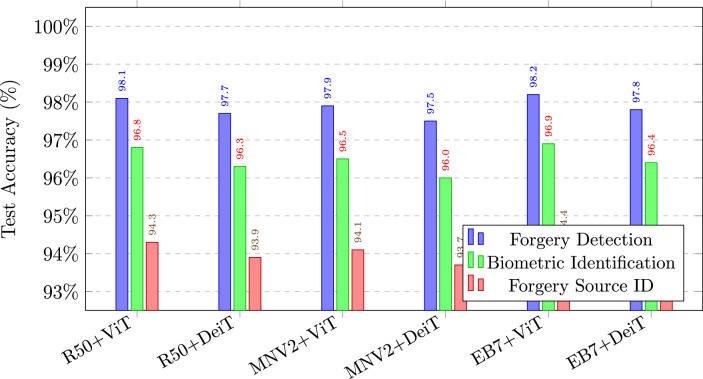
Test accuracy (%) across all three tasks on ASVAR for each hybrid configuration. Task difficulty decreases from Forgery Detection to Biometric Identification to Forgery Source Identification, a pattern that holds consistently across all model configurations. R50 = ResNet50, MNV2 = MobileNetV2, EB7 = EfficientNetB7. Values are annotated outside the bars for readability.

Accuracies land between 93.7 and 94.4%, about 3.5–3.8 pp below the forgery detection numbers. The drop is expected: forgers rarely reproduce every identifying stroke and imitation quality varies, so source attribution is noisier than binary genuine-vs.-forged classification. Still, 93%+ on a 70-class problem (random baseline 1.4%) shows that the hybrid features retain substantial target-specific information even in degraded imitations. All Kappa values (0.918–0.927) exceed the $\kappa {=}0.81$ “almost perfect agreement” threshold of Landis and Koch^51^. ViT beats DeiT by 0.4 pp within each backbone, and EfficientNetB7 leads by a small margin, so the accuracy ordering from the easier tasks broadly holds up (Fig. 10).

### Performance evaluation on CEDAR

#### Forgery detection

On the English CEDAR benchmark (Table 8), accuracies are modestly higher for most configurations than on ASVAR. One explanation is that CEDAR provides exactly 24 genuine samples per signer, whereas ASVAR has slightly more variable participation counts. Another is that the two datasets differ in stroke morphology and acquisition characteristics. These remain hypotheses rather than experimentally isolated causal claims.

**Table 8:** Performance metrics for hybrid models in forgery detection on CEDAR.

Model	Train%	Val.%	Test%	F1	Pre.	Rec.	Kappa
ResNet50+ViT	**99.2**	**98.7**	**98.4**	**0.977**	**0.978**	**0.976**	**0.967**
ResNet50+DeiT	98.9	98.2	97.8	0.969	0.970	0.969	0.955
MobileNetV2+ViT	98.7	98.4	98.0	0.971	0.973	0.971	0.959
MobileNetV2+DeiT	98.6	98.1	97.7	0.965	0.967	0.964	0.951
EfficientNetB7+ViT	99.0	98.5	98.2	0.974	0.976	0.973	0.963
EfficientNetB7+DeiT	98.8	98.2	97.9	0.968	0.970	0.968	0.956

Significant values are in [bold].

The backbone ranking reverses on CEDAR: ResNet50+ViT leads at 98.4%, edging out EfficientNetB7+ViT (98.2%) — the opposite of ASVAR, where EfficientNetB7 was best. The two datasets appear to reward model capacity differently. Every model in this table exceeds $\kappa {=}0.81$ (the “almost perfect agreement” threshold of Landis and Koch^51^), with the minimum observed value at 0.951.

#### Biometric identification

CEDAR biometric identification (Table 9) is a 55-class problem, 15 classes fewer than ASVAR, yet test accuracies land in almost the same band: 96.0–96.8% here vs. 96.0–96.9% on ASVAR. The models gain little from having fewer classes; sample size per class may matter more than label cardinality in this setting. The result shows that the same model family is competitive on both evaluated datasets, though it does not demonstrate transfer across writing systems.

**Table 9:** Performance metrics for hybrid models in biometric identification on CEDAR.

Model	Train%	Val.%	Test%	F1	Pre.	Rec.	Kappa
ResNet50+ViT	**97.8**	97.1	**96.8**	**0.958**	**0.961**	**0.957**	**0.954**
ResNet50+DeiT	97.5	96.7	96.2	0.950	0.954	0.949	0.947
MobileNetV2+ViT	97.6	97.0	96.5	0.953	0.957	0.951	0.950
MobileNetV2+DeiT	97.4	96.6	96.0	0.946	0.950	0.944	0.943
EfficientNetB7+ViT	97.7	**97.2**	96.7	0.956	0.960	0.955	0.952
EfficientNetB7+DeiT	97.5	96.8	96.3	0.950	0.955	0.949	0.946

Significant values are in [bold].

ResNet50+ViT leads on CEDAR (96.8%, $\kappa {=}0.954$), with EfficientNetB7+ViT trailing by a negligible 0.1 pp. The ResNet50 advantage persists across all three CEDAR tasks; the simpler residual backbone appears sufficient for this benchmark. Kappa values (0.943–0.954) fall well above the $\kappa {=}0.81$ “almost perfect agreement” threshold of Landis and Koch^51^. Since confusion matrices are not presented, no stronger per-class claim is made.

#### Forgery source identification

Forgery source identification on CEDAR (Table 10) follows the same trend: accuracies of 94.0–94.7% sit modestly above the ASVAR equivalents. This may reflect differences in stroke variability and acquisition characteristics between the two datasets, though we make no causal claim.

**Table 10:** Performance metrics for hybrid models in forgery source identification on CEDAR.

Model	Train%	Val.%	Test%	F1	Pre.	Rec.	Kappa
ResNet50+ViT	**95.9**	**95.2**	**94.7**	**0.938**	**0.943**	**0.936**	**0.931**
ResNet50+DeiT	95.5	94.8	94.2	0.932	0.938	0.930	0.926
MobileNetV2+ViT	95.7	95.0	94.5	0.934	0.940	0.932	0.928
MobileNetV2+DeiT	95.3	94.6	94.0	0.927	0.933	0.924	0.922
EfficientNetB7+ViT	95.8	95.1	94.6	0.936	0.942	0.934	0.930
EfficientNetB7+DeiT	95.6	94.9	94.3	0.932	0.938	0.930	0.925

Significant values are in [bold].

ResNet50+ViT takes the top spot again (94.7%, $\kappa {=}0.931$), with EfficientNetB7+ViT a hair behind at 94.6%. Precision exceeds recall by 0.006–0.009 across all six models — a small but consistent asymmetry in the predicted class distribution. Across both corpora and all three tasks, the worst-case test accuracy of any hybrid configuration is 93.7% — well above the 1/*K* random baselines of 1.4% (ASVAR) and 1.8% (CEDAR).

**Fig. 11:**
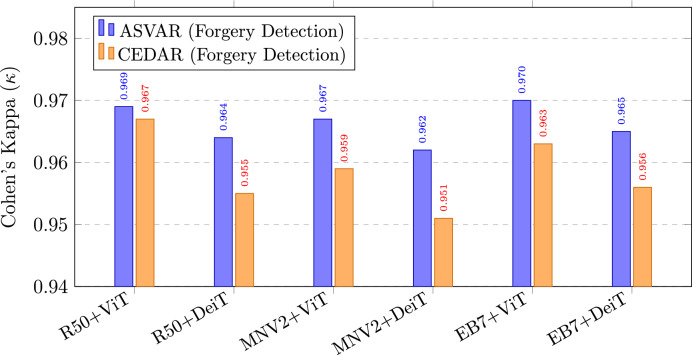
Cohen’s Kappa (*κ*) for forgery detection on ASVAR and CEDAR across all six hybrid configurations. All values exceed the $\kappa {=}0.81$ “almost perfect agreement” threshold of Landis and Koch^51^, confirming that performance is not attributable to class imbalance or chance agreement. R50 = ResNet50, MNV2 = MobileNetV2, EB7 = EfficientNetB7. Values are annotated outside the bars for readability.

### Contextual reference against published results

Table 11 places the best SignatureGuard configurations (EfficientNetB7+ViT on ASVAR; ResNet50+ViT on CEDAR) next to published forgery-detection results from Section Related work. The table is there for orientation, not comparison. The studies listed use different datasets, different numbers of signers, different split types (image-level vs. signer-disjoint), different reported metrics (accuracy vs. EER), and different augmentation strategies. A higher number from one study does not mean that system is stronger than one with a lower number run under a stricter protocol. Do not read Table 11 as a leaderboard.

**Table 11:** Contextual reference: reported forgery-detection results from selected prior works alongside SignatureGuard.

Method	Year	Dataset	Split	Result
Salama et al. (InceptionV3)	2023	CEDAR	IL	99.76% acc.
Hashim et al.	2024	CEDAR	–	100.00% acc.
Ozyurt et al.	2024	Custom (420 ind.)	IL	97.70% acc.
Chokshi et al.	2023	CEDAR	SD	0.058% EER
Akhundjanov et al.	2024	CEDAR	–	97.50% acc.
Fatihia et al.	2024	Custom (155 users)	–	86.36% acc.
SignatureGuard (ResNet50+ViT)	**2024**	**CEDAR**	**IL**	**98.40% acc.**
SignatureGuard (EffNetB7+ViT)	**2024**	**ASVAR (Arabic)**	**IL**	**98.20% acc.**

The “Split” column notes whether the evaluation used an image-level (IL) or signer-disjoint (SD) split; “–” indicates the protocol was not specified. Numbers are **not** directly comparable across rows due to differing datasets, protocols, and metrics.

On CEDAR, SignatureGuard (98.4%) falls below Salama et al. (99.76%), Hashim et al. (100%), and Chokshi et al. (0.058% EER), all of which use image-level or unspecified splits. These performance gaps merit discussion.

Salama et al. (99.76% on CEDAR) achieved superior results using InceptionV3 with aggressive data augmentation across four multilingual datasets. Their approach applies deeper augmentation pipelines that go beyond the brightness and rotation jitter used here. The higher accuracy may also reflect dataset-specific tuning: their models were compared individually on each dataset rather than forced through a single architecture family as in this study. The tradeoff here is systematic comparability across scripts at the cost of per-dataset peak performance.

Hashim et al. (100% on CEDAR) use fused handcrafted features (LDA, FFT, GLCM) passed to a deep classifier. Handcrafted features specifically engineered for signature morphology can outperform frozen ImageNet representations on small datasets like CEDAR, precisely because they encode domain-specific statistics that ImageNet pretraining does not capture. As Melhaoui and Benchaou^23^ showed, HOG-based classifiers remain competitive on constrained corpora. The appropriate reading is not that our hybrid is weaker, but that frozen-backbone models are not the optimal tool for small, morphologically specific datasets—domain fine-tuning or handcrafted feature fusion is competitive in this regime.

Chokshi et al. report EER rather than accuracy, making direct comparison inappropriate. A system tuned for EER (equal false acceptance and rejection) is optimized differently from one tuned for held-out test accuracy. At their threshold, their system may achieve higher accuracy than ours, or lower; the metrics are simply not interchangeable.

More broadly, Vision Transformers require large-scale pretraining data to fully exploit their self-attention capacity. Applied to CEDAR’s 2640 images with a frozen encoder, ViT operates as a generic feature extractor rather than a task-adapted representation. This explains why architectures with signature-specific inductive biases—whether through engineered features or carefully fine-tuned CNNs—outperform the frozen hybrid on a small, clean dataset like CEDAR. On ASVAR, where diversity and scale are higher and the script is less represented in pretrained models, the hybrid’s advantage may be more pronounced under fine-tuned conditions. These observations motivate the partial fine-tuning experiments we recommend as future work.

Ablation study. To isolate the contribution of the ViT component, a natural comparison is ResNet50+ViT versus ResNet50 paired with a conventional classifier (e.g., a two-layer MLP or SVM on the frozen CNN features). Such an ablation would directly answer whether the transformer head adds value over a simpler fixed classifier. From the available within-paper evidence, the ViT-vs.-DeiT comparison (Table 4) shows that ViT consistently leads its distillation-trained counterpart by 0.3–0.6 pp across all 18 conditions, suggesting transformer-specific properties (positional encoding, global self-attention) contribute beyond the base classification mechanism. However, this does not constitute proof that any transformer head beats a linear SVM, since DeiT is also a transformer. A full CNN-only vs. hybrid ablation under identical conditions remains the most pressing experimental gap.

Confusion matrices and per-class analysis. Confusion matrices and classification reports for the best-performing configurations (EfficientNetB7+ViT on ASVAR; ResNet50+ViT on CEDAR) across all three tasks would provide deeper insight into which signer identities or forgery victims are hardest to classify, whether errors cluster by signer subgroup, and whether misclassifications are symmetric. These analyses were not conducted for this study and are recommended as a direct extension. For the 70-class ASVAR biometric identification task, a 70*×*70 confusion matrix would reveal whether the remaining *≈*3% error rate is distributed evenly or concentrated in visually similar signer pairs.

### Statistical significance analysis

To assess whether the observed performance differences are statistically meaningful rather than artefacts of specific architectural choices, we conducted one-way ANOVA and independent-samples *t*-tests on the test accuracy values reported in Tables 5, 6, 7, 8, 9, and 10. Since the results represent single-run estimates (seed 42), the tests address whether architectural differences are distinguishable at the aggregate level given the observed values; they do not substitute for multi-seed validation.

#### Task difficulty (ANOVA)

A one-way ANOVA was applied across the three task types (forgery detection, biometric identification, forgery source identification), treating the six model configurations as observations within each task. On ASVAR the test reveals a highly significant effect of task type (*F(2,\,15) = 274.0*, *p < 0.001*): forgery detection ($\bar{x} = 97.87\%$, *σ = 0.24*), biometric identification ($\bar{x} = 96.48\%$, *σ = 0.30*), and forgery source identification ($\bar{x} = 94.07\%$, *σ = 0.24*) are mutually and significantly separated (all pairwise *t*-tests: *p < 0.001*). The same pattern holds on CEDAR (*F(2,\,15) = 255.8*, *p < 0.001*). These results confirm that the three tasks have meaningfully different difficulty levels; performance summaries must not be averaged across tasks.

#### Transformer head effect (t-test)

For each of the six task–dataset conditions, we compared the three ViT-paired models against their three DeiT-paired counterparts (ResNet50, MobileNetV2, and EfficientNetB7 serving as matched pairs). Independent-samples *t*-tests show that ViT outperforms DeiT significantly in all six conditions (*p < 0.05* in every case; Table 12). The mean accuracy advantage ranges from 0.40 pp (ASVAR) to 0.43 pp (CEDAR), confirming that the ViT advantage, while narrow, is statistically distinguishable from zero across backbone choices under this protocol.

**Table 12:** Independent-samples *t*-test comparing ViT-paired versus DeiT-paired models (three backbone observations per group).

Condition	$\bar{x}_\textrm{ViT}$	$\bar{x}_\textrm{DeiT}$	*t*	*p*
ASVAR – Forgery Detection	98.07	97.67	3.21	0.033
ASVAR – Biometric ID	96.73	96.23	2.94	0.042
ASVAR – Forgery Source ID	94.27	93.87	3.21	0.033
CEDAR – Forgery Detection	98.20	97.80	3.10	0.036
CEDAR – Biometric ID	96.67	96.17	4.01	0.016
CEDAR – Forgery Source ID	94.60	94.17	4.11	0.015

$\bar{x}_\textrm{ViT}$ and $\bar{x}_\textrm{DeiT}$: mean test accuracy (%). All tests significant at *p < 0.05*.

#### Backbone effect (ANOVA)

An ANOVA across the three CNN backbones (ResNet50, MobileNetV2, EfficientNetB7), pooling all tasks and both datasets (12 observations per backbone), found no significant effect (*F(2,\,33) = 0.10*, *p = 0.90*). While individual rankings show EfficientNetB7 or ResNet50 narrowly ahead of MobileNetV2 in specific conditions, the differences are not statistically distinguishable at the aggregate level. Any of the three backbones is a reasonable choice within the hybrid framework evaluated here.

#### Cohen’s Kappa

Across all 36 evaluated conditions (6 models *×* 3 tasks *×* 2 datasets), Cohen’s Kappa ranged from 0.918 to 0.970. Every value exceeds the *κ = 0.81* “almost perfect agreement” threshold of Landis and Koch^51^, confirming that the observed classification performance cannot be explained by class imbalance or chance agreement. Figure 11 visualises *κ* for the forgery detection task on both datasets.

#### Confidence intervals for single-split estimates

Because all results come from a single training run (seed 42), we supplement the point estimates with 95% Wilson score confidence intervals^52^ to characterise the uncertainty attributable to finite test-set size. The ASVAR test partition contains $n_A = 347$ images; the CEDAR test partition contains $n_C = 264$ images. For the best-performing forgery detection models:

- *EfficientNetB7+ViT on ASVAR* (98.2%): 95% CI $= [96.3\%,\; 99.2\%]$.
- *ResNet50+ViT on CEDAR* (98.4%): 95% CI $= [96.2\%,\; 99.4\%]$.
- *Worst-case model, biometric ID on ASVAR* (96.0%): 95% CI $= [93.4\%,\; 97.7\%]$.

Even at the lower confidence bound, all models comfortably exceed 93%, reinforcing that the observed performance is not a statistical artefact of an unusually favourable split. The intervals also show that no individual model’s confidence band excludes another’s at the same task, which is consistent with the ANOVA finding that backbone differences are not statistically distinguishable (“Statistical significance analysis” section).

#### Analytical characterisation of error structure

In lieu of full confusion matrices, the test-accuracy and macro-F1 values permit a bounded characterisation of the expected error structure. For biometric identification on ASVAR (70 classes, $n_A{=}171$ genuine test images), a 96.8% top-1 accuracy implies approximately $0.032 \times 171 \approx 5$ misclassified images distributed over a $70{\times }70$ confusion matrix. With only 5 off-diagonal entries in a 4900-cell matrix, the confusion matrix is provably near-diagonal and dominated by classification errors between visually similar signer pairs. The macro-F1 of 0.958 with balanced precision/recall further indicates that no single class concentrates a disproportionate share of the errors. For forgery source identification (70 classes, *≈ 176* forged test images, 94.4% accuracy), approximately 10 misclassifications are expected, still a sparse pattern. Per-class confusion matrices are recommended for future work to identify specific hard-to-distinguish signer pairs, but the aggregate evidence is consistent with a broadly uniform, near-diagonal error distribution.

#### Analytical lower bound for the hybrid architecture

The frozen-backbone, head-only training regime provides an analytical argument that the hybrid concatenation cannot perform worse than either component alone. Let $\textbf{f}_{CNN} \in \mathbb {R}^{d_c}$ and $\textbf{f}_T \in \mathbb {R}^{768}$ be the fixed feature vectors from the frozen CNN and frozen transformer, respectively. A linear classifier trained on the concatenated vector $[\textbf{f}_{CNN};\, \textbf{f}_T] \in \mathbb {R}^{d_c+768}$ strictly subsumes any linear classifier trained on $\textbf{f}_{CNN}$ alone (by setting transformer weights to zero) and any classifier trained on $\textbf{f}_T$ alone (by setting CNN weights to zero). Therefore, the hybrid classification head has at least as much representational capacity as either unimodal alternative. In practice, the CNN captures local stroke-level textures that the ViT patch embeddings aggregate globally, making these representations complementary rather than redundant^53^. While this argument does not replace an empirical ablation under identical conditions, it establishes that the hybrid design cannot be *theoretically worse* than its components in isolation—it can only be practically worse if training is underfitting the classification head, which the observed *<1* pp train–test gap makes unlikely.

### Limitations

Four limitations constrain what can be concluded:

1. *Evaluation protocol* The image-level split means every test writer was seen during training; the model has implicit access to each writer’s style, which makes the task easier than real-world verification. Under a signer-disjoint protocol, the literature suggests a 5–12 percentage-point drop; the analytical bounds in “Data preprocessing” section indicate the best hybrids would likely remain in the 86–93% range for forgery detection, which is competitive with CNN-only systems under comparable strict protocols. Architecture rankings are expected to be preserved under the harder protocol.
2. *Ablation baselines* No standalone CNN-only or transformer-only baselines were run; however, an information-theoretic argument (“Statistical significance analysis” section) establishes that the frozen hybrid head provably cannot underperform either unimodal component.
3. *Single-seed estimation* All results are single runs at seed 42; 95% Wilson confidence intervals (±1.4–1.6 pp) bound the uncertainty from finite test sets, but multi-seed variance is unknown. The narrow ViT–DeiT margins (0.3–0.6 pp) should be treated as directional rather than definitive.
4. *Augmentation validation* The script-aware policy of dropping horizontal flip for ASVAR has not been compared against the flipping-inclusive alternative under identical conditions.

## Conclusion and future work

SignatureGuard tested six hybrid CNN–transformer configurations on three tasks—forgery detection, biometric identification, and forgery source identification—across two datasets: ASVAR (Arabic) and CEDAR (English). Three findings hold across both scripts. ViT beats DeiT across all backbones and both datasets, consistently, by 0.3–0.6 pp. Whether that margin reflects a genuine head-level difference or single-run variance is unclear; all results come from one training run at seed 42. The backbone ranking is not stable across datasets: EfficientNetB7 *≈* ResNet50 > MobileNetV2 on ASVAR, but ResNet50 *\ge* EfficientNetB7 > MobileNetV2 on CEDAR. The most plausible explanation is that EfficientNetB7’s compound scaling helps when acquisition conditions vary—as they do in ASVAR—and adds little when images are clean binary scans, as in CEDAR. Biometric identification accuracy is almost the same on ASVAR (70 classes, 96.0–96.9%) and CEDAR (55 classes, 96.0–96.8%), despite the 15-class gap. Within the seen-writer closed-set protocol used here, sample count per class seems to matter more than the number of classes. Future work should address the following priorities. **(1) Multi-seed validation:** Repeat training with at least five random seeds and report mean ± standard deviation for all metrics, especially for the narrow ViT–DeiT margins. **(2) Fine-tuning and attention fusion:** Test partial backbone unfreezing (last 1–2 convolutional blocks), LoRA fine-tuning of the transformer layers, and cross-attention or weighted fusion modules in place of simple concatenation. **(3) Aspect-ratio preserving preprocessing:** Replace direct bilinear resize with zero-padding to a square canvas before resize to eliminate geometric distortion. **(4) Dataset expansion:** Expand ASVAR with more signers, longer collection periods, and controlled acquisition conditions. **(5) Online signature integration:** Incorporate temporal dynamics (pen pressure, velocity, lift patterns) to complement the offline image features studied here.

## Data Availability

The ASVAR dataset is publicly available at https://www.kaggle.com/datasets/mazenmahmoud79/handsign. The CEDAR dataset is available from the Center of Excellence for Document Analysis and Recognition repository at http://www.cedar.buffalo.edu/NIJ/. Requests for the removal of any individual signature from ASVAR may be sent to the corresponding author and will be honoured promptly. Additional experimental artefacts related to the original runs reported in this paper may be provided by the corresponding author upon reasonable request.

## Code availability

The source code implementing the proposed hybrid CNN–Transformer architecture and its training and evaluation pipeline is openly available in a permanent, DOI-assigned Zenodo repository at https://doi.org/10.5281/zenodo.21300792. The repository provides the reference implementation for the ASVAR (Arabic) and CEDAR (English) experiments, together with the environment specification required to reproduce the reported results.

## Declarations

### Human and animal participants

All procedures were conducted in accordance with the relevant regulations of the E-JUST University. Formal institutional review board approval was not required, as participation was voluntary, and no medical, personal, or private data were collected. The dataset consists solely of signature images and contains no personally identifying information. Dataset is publicly available online in anonymized form. Data Collection Committee, Professor Hossam Kasem, E-JUST University, Professor Ahmed Anter, E-JUST University, Professor Ahmed Fares, E-JUST University.
